# Association Between the Enriched Environment Level and Serum Brain-Derived Neurotrophic Factor (BDNF) in Patients with Major Depressive Disorder

**DOI:** 10.3390/brainsci14111137

**Published:** 2024-11-13

**Authors:** Andrés Vega-Rosas, Mónica Flores-Ramos, Gerardo Bernabé Ramírez-Rodríguez

**Affiliations:** 1 Laboratorio de Epidemiología Clínica, Subdirección de Investigaciones Clínicas, Instituto Nacional de Psiquiatría Ramón de la Fuente Muñiz, Calzada México-Xochimilco #101, Col. San Lorenzo Huipulco, Tlalpan, Mexico City C.P. 14370, Mexico; dr.andresvegar@gmail.com; 2 Laboratorio de Neurogénesis, Subdirección de Investigaciones Clínicas, Instituto Nacional de Psiquiatría Ramón de la Fuente Muñiz, Calzada México-Xochimilco #101, Col. San Lorenzo Huipulco, Tlalpan, Mexico City C.P. 14370, Mexico; gbernabe@inprf.gob.mx

**Keywords:** Major Depressive Disorder, environmental enrichment, Brain-Derived Neurotrophic Factor (BDNF)

## Abstract

Major Depressive Disorder (MDD) is a neuropsychiatric condition whose neurobiological characteristics include alterations in brain plasticity, modulated by Brain-Derived Neurotrophic Factor (BDNF). In animal models, environmental enrichment promotes neuroplasticity and reduces depressive-like behaviors. In humans, we proposed to assess the level of Enriched Environment (EE) using a questionnaire that includes different domains of the EE (cognitive, social, and physical), which we named the EE Indicator (EEI). Objective: To determine the relationship between the level of EE and serum BDNF in participants with MDD and healthy controls. Materials: Participants with MDD without antidepressant treatment and healthy controls were recruited, and their EE level and serum BDNF concentration were determined looking for correlations between their clinical characteristics and the cognitive, social, and physical activities according to the EEI. Results: A total of 25 participants were recruited, of which 6 participants with MDD and the same number of controls were selected in a paired manner. Although no differences were found in the concentration of BDNF between the groups, positive correlations were observed between cognitive EE and BDNF (r = 0.62, *p* = 0.035), as well as negative social EE and the Hamilton Depression Rating Scale (HDRS) (r = −0.86, *p* = 0.001). The sum between cognitive and social EE showed a positive correlation with the serum concentration of BDNF (r = 0.34, *p* = 0.0451). Conclusions: The level of EE is potentially modulating the presence and severity of MDD at a clinical level, but it can also influence at a neuroplastic level through promoting or limiting the concentration of BDNF.

## 1. Introduction

Major Depressive Disorder (MDD) is a multifactorial neuropsychiatric condition with neurobiological and psychopathological characteristics. Although genetics and alterations in the central nervous system (CNS) at the neuroplastic, immunological, and neurotransmitter levels play an important role [1,2], the environment and life experiences will be determining factors for the development of MDD [3]. One of the main actors responsible for modulating neuroplasticity in adults is the Brain-Derived Neurotrophic Factor (BDNF) [4], and it has been seen that in preclinical models associated with depression [5,6,7] such as chronic stress [8,9,10] there is a decrease in the central and serum concentration of BDNF, down-regulating neural plasticity, promoting neuronal atrophy, and decreasing synaptic function, expressed in its number of apical dendrites and its total dendritic spines, structures intimately involved with cognitive processes [11]. Nevertheless, associating serum BDNF level with MDD or its severity remains controversial [12,13], as not all studies have demonstrated a significant difference at pretreatment baseline.

**Figure 1:**
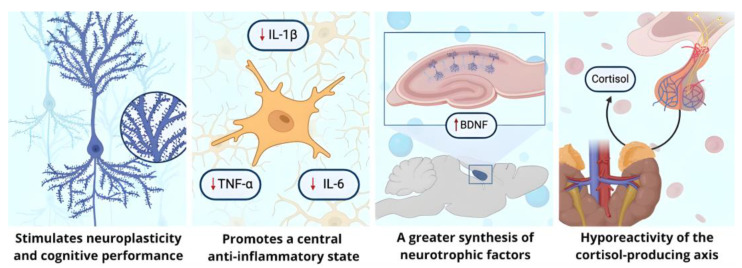
Neurobiological effects associated with environmental enrichment. In different animal models of environmental enrichment, this type of paradigm has been associated with a promotion of neuroplasticity accompanied by an improvement in the performance of cognitive tasks, a change in the microglial activation profile for a lower synthesis of proinflammatory cytokines such as interleukins related to depression and stress, an increase in the synthesis and expression of neurotrophic factors (e.g., BDNF), and a decrease in the production of catecholamines due to a lower reactivity of the hypothalamus–pituitary–adrenal axis. Created with BioRender.com. (URL: https://www.biorender.com/ accessed on 19 September 2024).

On the other hand, environmental enrichment provides external stimuli that favor the development of adaptive behaviors in humans and animals [14,15,16], improving reactivity to stress, promoting neurogenesis and neuroplasticity, increasing expression of neurotrophins such as BDNF, generating an anti-inflammatory profile, and decreasing activation in the hypothalamic–pituitary–adrenal axis, as well as in the sympathetic tone [17,18,19] (Figure 1). Various of these protocols have been tested in rodent models that cover the social, cognitive, and physical activity domains, which individually have proven to improve depressive-like symptoms and increase BDNF concentrations, but the combined implementation of these domains promotes a more robust effect [20,21,22].

Therefore, the basal environment in which an individual is located may mediate mood as a potential risk or protective factor for developing MDD and other neuropsychiatric disorders [23,24,25]. However, this hypothesis has also been proved in preclinical studies [26]. Therefore, exploring the implications of the Enriched Environment (EE) level, independently of interventions aimed at enrichment by a third party, allows an approximation to the basal state. To have an objective parameter of the EE of patients with MDD, we previously proposed an EE Indicator (EEI), which evaluates and generates a score based on the frequency of engaging in enriching activities across cognitive, social, and physical activity domains while also considering the subjective enjoyment of these activities [27]; this scale has been validated and standardized in its Spanish version for the Mexican population.

The EEI allows the scores of the cognitive and social domains in points, and the physical exercise measured by Metabolic Index Units (METs), to be added to give rise to a score with which a person’s EE level can be classified as high, moderate, or low. While the EEI proved to be valid for use in clinical settings and allows inferring the level of EE according to daily activities and the subjective enjoyment they generate in healthy people and MDD patients, it has presented controversies and specific limitations for the evaluation of physical activity. Still, it represents an underexplored area of opportunity to comprehensively evaluate EE in humans. Then, we hypothesized that higher peripheral concentrations of BDNF positively correlate with a higher EEI, but in MDD patients, BDNF and EEI will be lower than in healthy control subjects (CSs). Thus, this pilot study aimed to evaluate the presumed relationship between the level of EE and the serum concentration of BDNF in participants diagnosed with MDD compared with CSs.

## 2. Materials and Methods

### 2.1. Participants

**Figure 2:**
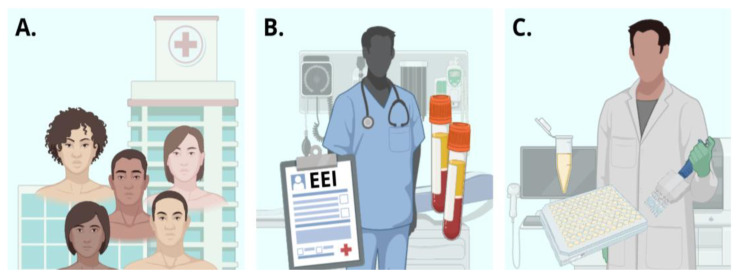
Participant recruitment, data collection and analysis. (**A**) Participants diagnosed with Major Depressive Disorder and control subjects were recruited. (**B**) A medical evaluation was performed with application of the Enriched Environment Indicator and clinical laboratory tests. (**C**) Determination of serum levels of BDNF by ELISA and data analysis.

Participants with MDD were recruited from the first psychiatric evaluation area at a third-level psychiatric hospital in Mexico City (Figure 2). The clinical diagnosis of MDD was established in accordance with the Diagnostic and Statistical Manual of Mental Disorders, fifth edition (DSM-5) criteria, supported by a score of no less than 13 points in the 17-item Hamilton Depression Rating Scale (HDRS). MDD participants were not taking any psychopharmacological treatment at the time of the interview. For the healthy CSs, we considered people who had not sought psychiatric care, who were in adequate physical health and who obtained a score of less than 7 HDRS points in their evaluation. All participants were free of any medication and did not have a diagnosis of other medical illness, including substance abuse.

Clinical interviews were conducted by a trained physician considering inclusion criteria and ruling out the possibility of psychiatric comorbidities; the Mini Neuropsychiatric Interview (MINI) was applied in the CSs to rule out any psychiatric condition. In addition, the questionnaire of the Mexican Association of Market Intelligence and Opinion Agencies (AMAI 2022) was applied to determine socioeconomic level in all participants.

Only adults over 18 years of age were recruited, to whom the present protocol was explained, and after they decided to accept participation, they signed the informed consent approved by the Ethics and Research Committees of the Instituto Nacional de Psiquiatría Ramón de la Fuente Muñiz (CEI/C/010/2022).

### 2.2. Assessment Procedure

Demographic information such as gender, age, schooling, marital status, and employment situation was assessed by the evaluating physician at the time of recruitment. Through the clinical laboratory of the same hospital, lipid and thyroid profiles, morning glucose and cortisol, and testosterone (free and total) were determined, and gynecological hormonal profile was added in women. For MDD participants, the age of illness onset, illness evolution and duration in weeks, severity of the current condition at the time of evaluation, recent suicidal ideation, and psychotherapy treatments were interrogated.

### 2.3. Environmental Enrichment Indicator

To determine the EE load as a quantitative score, the EEI [27] was applied. This instrument is a test that explores the quantity, frequency of activities people typically engage in, and subjective satisfaction. Examples of the questions to evaluate the domains of the EEI are:

- Cognitive, e.g., playing chess, reading, home repairs, cooking new recipes.
- Social, e.g., family life, interaction on social media, hanging out with coworkers.
- Physical exercise, evaluating the activities by intensity, duration, and frequency.

Each activity obtains different scores according to the frequency with which it is carried out. For each domain, its score is obtained and its level is determined as low, moderate, or high, based on the cut-off point marked by the instrument. To obtain the overall result of the EE level, the following rule is used:

- Low: 2 or 3 domains obtained in the low level.
- Moderate: 2 or all domains obtained as moderate, or one domain in each level.
- High: 2 or all domains obtained in the high level.

### 2.4. Serum Samples and Quantification of BDNF

Blood samples were obtained using a standardized hospital protocol. The samples were centrifuged to separate the serum, which was then stored at −80 °C for further analysis. Determination of BDNF levels was carried out using a Human Free BDNF Quantikine enzyme-linked immunosorbent assay (ELISA) kit, following the instructions provided by the manufacturer (DB00; R&D Systems, Minneapolis, MN, USA; catalog number of the kit DY248, lot P284236; range 1500–23.4 pg/mL). Plates were read with a Glomax Discover microplate reader (Promega, Fitchburg, WI, USA). The overall protein concentration in the centrifuged serum samples was determined using the Bradford test, for which the samples were diluted 1:15 with the kit’s diluent reagent, according to the manufacturer’s standardized parameters. The optical density of each sample was read in triplicate at 600 nm, to average it and obtain the final concentration of each participant.

### 2.5. Statistical Analysis

For sample description, frequencies and percentages for categorical variables and means and standard deviations (SD) for continuous variables were used. Demographic features were compared between groups using chi-square tests for categorical variables and independent-sample t-tests for the comparison of continuous variables. Linear regression analysis was performed to determine the association between HDRS score and BDNF concentration. Clinical and paraclinical variables were analyzed using a Spearman correlation matrix to determine associations. Comparison of serum BDNF and cortisol concentrations from MDD participants and healthy controls was performed using the nonparametric Kruskal–Wallis test. To evaluate if the participants were appropriately categorized into subgroups depending on their level of EE, a one-way ANOVA test was performed with the total sum of the EEI score, as well as a breakdown score for each domain: social, cognitive, and physical activity. The level of statistical significance was set at *p* ≤ 0.05. All statistical analyses were conducted using GraphPad Prism software version 9.5.1 for macOS (La Jolla, CA, USA).

## 3. Results

### 3.1. Sample Description and Peripheral BDNF Concentrations

A total of 25 participants were recruited, of which 19 were patients with a confirmed MDD diagnosis (men *n* = 4, women *n* = 15) and 6 were CSs (men *n* = 3, women *n* = 3). Six participants with MDD and six CSs were selected in a paired manner based on sex, age, and general characteristics. Additionally, each group was analyzed according to the score they obtained in the EEI: (low or medium; no MDD or CS participants who were recruited met high-EE criteria) with *n* = 3 for each group (Table 1). Unfortunately, we could not recruit participants qualifying for high-EE criteria.

All participants belonged to the same age group with an average of 26.75 ± 2.87 years, with the same ratio in the proportion of men and women, and without differences between their socioeconomic status (MDD = 150.3 ± 38.22 points vs. CSs = 163.3 ± 31.23 points of AMAI scores, *p* = 0.5334). They presented a frankly different HDRS score (*p* < 0.0001), corroborating the clinical congruence with the groups where the participants were categorized as MDD or CS. Serum BDNF levels in CSs showed a tendency to be higher than in participants with MDD, although they did not reach statistical significance (Table 1).

The possible relationship between the HDRS score, which illustrates the severity of MDD, and serum levels of BDNF was evaluated; however, no significant association was found when analyzing the entire sample (*p* = 0.1318). The groups were evaluated separately through linear regressions, however, neither the CS (*p* = 0.3876) nor the MDD group (*p* = 0.0740) were significantly related to BDNF levels. Despite this, there is a tendency that the higher the score on the HDRS, the lower the serum concentration of BDNF.

### 3.2. Correlations Among Variables

**Figure 3:**
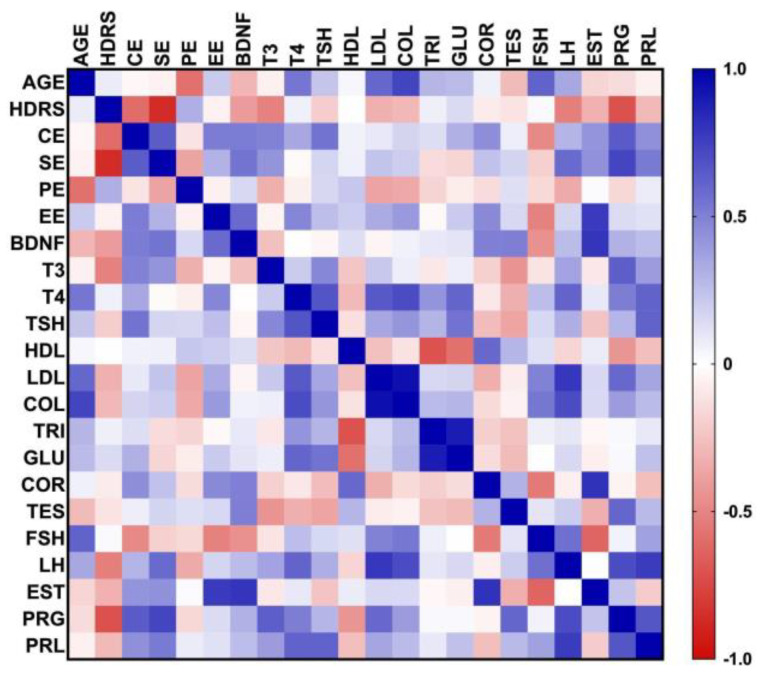
Correlation matrix of the clinical variables, BDNF, and EEI domains. Abbreviations: Hamilton Depression Rating Scale (HDRS), Cognitive Environment (CE), Social Environment (SE), Physical Environment (PE), Enriched Environment level (EE), Brain-Derived Neurotrophic Factor (BDNF), triiodothyronine (T3), thyroxine (T4), Thyroid Stimulating Hormone (TSH), High Density Lipoproteins (HDL), Low Density Lipoproteins (LDL), Total Cholesterol (COL), Triglycerides (TRI), Glucose (GLU), Morning Cortisol (COR), Total Testosterone (TES), Follicle Stimulating Hormone (FSH), Luteinizing Hormone (LH), Estradiol (EST), Progesterone (PRG), Prolactin (PRL). The blue color refers to directly proportional relationships and red to inversely proportional ones; the more saturated the color, the greater the strength of association and statistical significance.

A global Pearson correlation matrix (Figure 3) was performed to determine associations between age, HDRS score, serum BDNF concentration, and clinical laboratory results (partial thyroid profile, lipid profile, fasting glucose, morning cortisol, testosterone, and gynecological profile), as well as EE level in total score and broken down by domains: Cognitive Environment (CE), Social Environment (SE), and Physical Environment (PE). The variables that have a significant positive or negative correlation are listed in Table 2. The way EEI behaves with the serum concentration of BDNF stands out as having a positive correlation (*p* = 0.627). However, it is interesting how the sum of CE and SE gives rise to a synergistic effect that reaches significance, instead of when the domains are analyzed separately. Regarding the negative correlation between the SE and the HDRS score, it refers to how a participant with MDD will mainly have a condition in their social interactions frequently manifested as isolation, a clinical manifestation adequately verified from the interpretation of the EEI. In the case of the significant correlations referring to paraclinical laboratories, their associations are explained based on the physiological role they play in a manner already well known in the medical literature.

### 3.3. Variations in BDNF Concentrations According to the Different Domains of Enriched Environment

**Figure 4:**
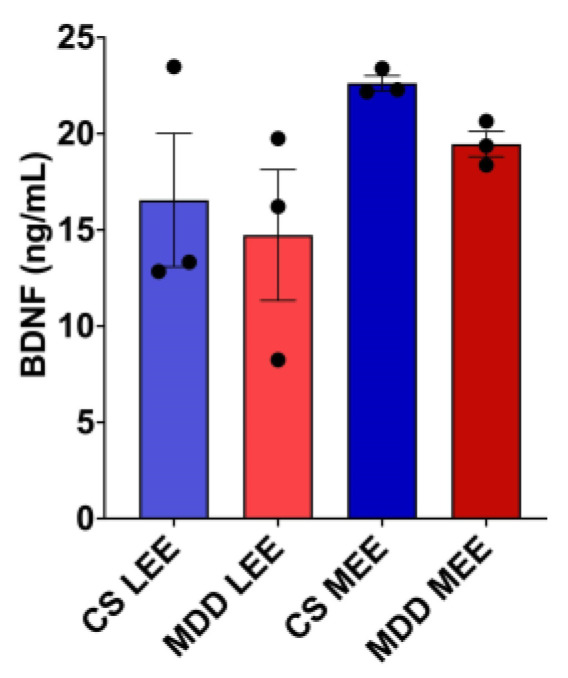
Comparison between serum BDNF according to the level of Enriched Environment. Average concentrations and abbreviations: Control Subjects with Low Enriched Environment (CS LEE) = 16.55 ± 6.0, MDD Participants with Low Enriched Environment (MDD LEE) = 14.73 ± 5.9, Control Subjects with Medium Enriched Environment (CS MEE) = 22.625 ± 0.6, MDD Participants with Medium Enriched Environment (MDD MEE) = 19.46 ± 1.1. One-way ANOVA *p* = 0.2401.

The means of BDNF levels in the different subgroups according to their total EE were compared with no significant differences (*p* = 0.2401); again, the controls showed a trend toward higher serum BDNF concentrations than their respective comparisons with the participants with MDD (Figure 3). In addition, in participants with MDD, the mean is increased when comparing a low versus medium EE level, showing a trend that the higher the EE level, the greater the BDNF (Figure 4).

**Figure 5:**
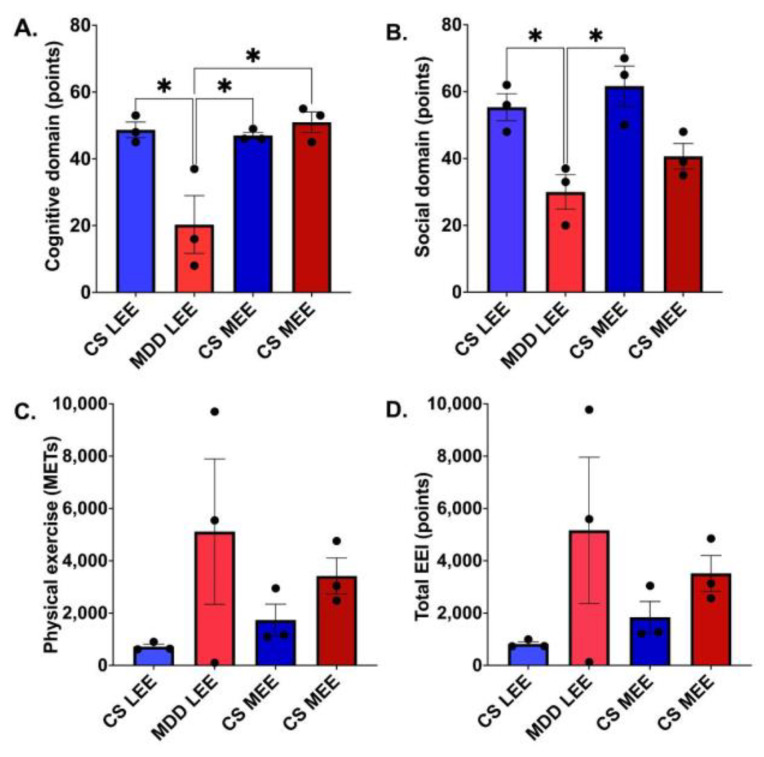
Confirmation of classification of participants by EE level. (**A**) Cognitive domain (*p* = 0.0058); (**B**) Social domain (*p* = 0.0066); (**C**) Physical exercise (*p* = 0.2355); (**D**) Total Environmental Enrichment Indicator (EEI) score (*p* = 0.2464). Abbreviations: Control Subject with Low Level of Enriched Environment (CS LEE), Participant MDD with Low Level of Enriched Environment (MDD LEE), Control Subject with Medium Level of Enriched Environment (CS MEE), MDD Participant with Medium Level of Enriched Environment (MDD MEE). Each point represents a participant. Asterisks mean *p* ≤ 0.05.

After determining that there were no significant differences between the BDNF of the different groups classified according to their level of total EE, it was decided to break down the results of the EEI into its different domains: CE, SE, and PE. To determine the clinimetric differences between the different domains of the EEI, the participants were analyzed into subgroups depending on their level of EE. A Bonferroni test was performed with the total sum of the EEI points, as well as the points of each area individually (Figure 5), finding significant differences (Table 3).

**Figure 6:**
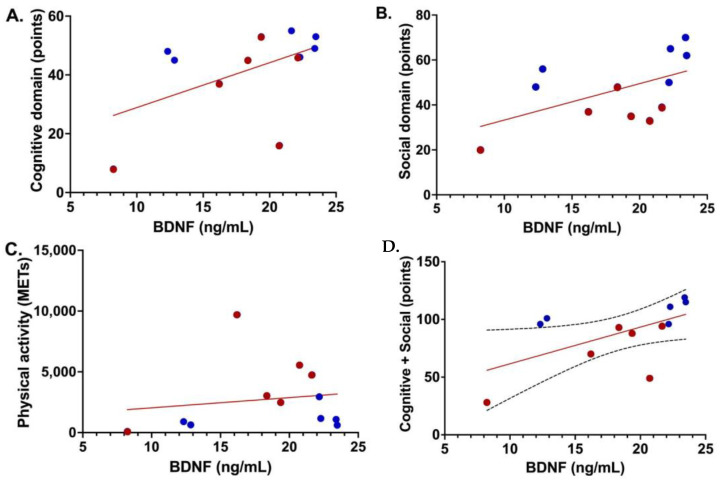
Association between the level of EE and BDNF broken down by domains. (**A**). Cognitive domain: *p* = 0.066. (**B**). Social domain: *p* = 0.066. (**C**). Physical activity: *p* = 0.64. (**D**). Cognitive domain + Social domain r = 0.34, *p* = 0.0451. Red dots represent MDD participants and blue dots represent CS participants.

When analyzing each domain separately (CE, SE and PE), no significant associations were found with the BDNF (CE: *p* = 0.066, SE: *p* = 0.066, and PE: *p* = 0.64), but when adding CE + SE, we found a synergistic effect that positively correlated with serum BDNF levels (*p* = 0.0451), as seen in Figure 6.

## 4. Discussion

Numerous studies have proposed BDNF as a critical molecule in the dynamic changes in brain plasticity in mammals, which is negatively affected during MDD in humans as well as in animal models of stress, and which can be increased in its serum concentration by EE [17]. However, similar to what has been reported in the literature where the data are controversial [28], participants with MDD in this protocol presented a lower average serum BDNF compared to healthy controls, although not significantly. In this same sense, significant changes in serum BDNF concentrations have been found, especially when participants are subjected to pharmacological antidepressant treatments only with certain selective serotonin reuptake inhibitors or when undergoing electroconvulsive therapy [5,29]. However, this difference is not found in a homogeneous and replicable way in all psychotropic drugs, so finding this non-significant difference in the present sample is in accordance with what has been reported in other studies that analyze serum BDNF in humans.

Among the main tools used in clinical practice for the diagnosis and determination of severity of MDD, the HDRS is used [30]. It was found that the score of this scale tends to have a negative correlation with the serum concentration of BDNF, so that the greater the severity of the symptoms and dysfunctions of MDD represented by a higher score on the HDRS, the lower the concentrations of serum BDNF.

When classifying participants into the different groups according to their EE level, in controls and participants with MDD no differences were found regarding their concentration of BDNF, since when breaking down the domains (CE, SE, and PE domains), a significant dispersion was found in the scores of physical EE. When interviewing participants with MDD, some report a large amount of physical activity in their work or transportation activities that was not subjectively enjoyable, which increases the dispersion of the data and does not necessarily offer the same benefits as regular physical training. In animal models, it has been questioned which domain of EE is most important to promote neuroplasticity or reduce depressive/anxious behaviors [31], so it is convenient to analyze it as a whole through the EEI, and by domains.

In murine models of dementia with an intervention of environmental enrichment, it was seen that the groups that received enrichment only in the PE domain experienced a smaller effect on anxiety-related behavior and risk assessment behavior deficits in the Alzheimer’s disease models [32]. When exploring models of neuroimmune mechanisms during normal aging, in the short-term, CE is a stronger modulator of microglial and peripheral T cell subset numbers than PE [33]. However, when comparing the effects of forced exercise training and voluntary physical activity in rats under an Enriched Environment protocol, in both cases hippocampal neuroplasticity improved and BDNF levels increased, although this was more pronounced in those that were not forced; similar results were reported for vascular endothelial growth factor (VEGF) [34]. Consequently, the subjective enjoyment of activities and the enriched domain can generate differences between the effects obtained at the neurobiological and behavioral levels.

Independently analyzing the EE score in the social domain, it was found that it is correlated with the concentration of BDNF, such that the greater the number of social interactions with their respective subjective enjoyment, the higher the concentrations of this neurotrophin in the blood. Social EE could not only mediate the production of BDNF and an anti-inflammatory profile in the hippocampus, but also the presence of the diagnosis of MDD, as well as its severity [35].

It is well known that social support networks help promote mental health and prevent its different disorders, especially MDD, in the same way these support networks encourage better adherence to treatment and favor the prognosis within MDD, so corroborating this basic clinical integration between social interactions and their influence on the clinical and neurobiological changes of MDD is of great value to be considered in preventive actions and in multidisciplinary treatment schemes [36,37]. Similarly, social interactions have been shown to be one of the areas of EE with the greatest impact in reducing depressive-type behaviors in animal models subjected to chronic stress or social isolation [38,39,40], in addition to promoting optimal resolution in cognitive tasks, especially memory.

Although the mechanisms by which environmental enrichment can increase BDNF concentration are not yet fully defined, advances point to the possibility that it may be through epigenetic regulation. Upregulation and methylation of exon IV of the gene encoding BDNF in the prefrontal cortex has been proposed, which has also been shown to be deficient in stress-related psychopathologies [41].

In different animal studies, an improvement in depressive-type behaviors or changes associated with neuroplasticity and neuronal metabolism have been seen after exposure to EE paradigms [21,42,43]. However, these results become truly consistent when animals are given stimuli in more than one domain, in such a way that those that only have cognitive EE can improve task resolution, but not with the same effectiveness if social or physical EE is added at the same time. The sum of the social and cognitive EE scores was analyzed, excluding the physical activity score due to the previously mentioned dispersion, and it was found that in a linear regression they have a statistically significant relationship with the serum concentration of BDNF, so that EE may be modulating serum neurotrophic factors in MDD.

Measuring how enriching physical activity is through information subjectively declared by participants is a limitation found in the original EEI standardization [27], compared to the cognitive and social domains. Proposing objective methods to determine the impact of activities in this domain would allow the instrument to be more precise and in line with what is well known about the production of BDNF by striated muscle during physical exercise [44,45,46]; this peripheral production of BDNF is a possible explanation of how environmental enrichment promotes neuroplasticity.

Implementing environmental enrichment strategies in humans has many limitations: subjecting the participant to a completely controlled environment for a certain time, the subjectivity that people have to enjoy hedonically when carrying out enriching activities, and the erroneous idea that EE is synonymous with a higher socioeconomic status. However, we did not notice this effect and we have not found any study that addresses it outside of childhood [47], when neurodevelopment is active and is influenced by the structural and functional changes.

The main limitation of the present study was the sample size, which turned out to be small due to the short time available to recruit patients for this pilot study. On the other hand, the report on enriching activities’ performance is based on the participants’ declarative information. No objective methods were used to corroborate the information obtained; implementing technological tools that provide objective data could improve the analysis of a load of enriching activities beyond the participants’ subjective perception.

Analyzing a statistically significant population would not only allow us to strengthen the evidence found and confirm the findings but would also offer the opportunity to apply environmental enrichment as part of a comprehensive therapeutic strategy for mental health problems, such as MDD.

Future studies could consider other biomarkers of Enriched Environment with an impact on the cognitive and behavioral area. In young senescence-accelerated-prone mice, neurotrophic changes have been seen in pleiotrophin (PTN) in addition to BDNF, or neurodegeneration such as GSK3, amyloid-beta precursor protein, and phosphorylated beta-catenin [48]. In the case of humans with neurological disorders, rather than using biomarkers, the impact of environmental enrichment on clinical recovery processes continues to be evaluated [49].

## 5. Conclusions

The data from this pilot study allows us to infer that the level of EE potentially modulates the presence and severity of MDD at a clinical level, especially when considering the sum of the social and cognitive domains. Still, it can also have an influence at a neuroplastic level by promoting the concentration of serum BDNF. Finally, our findings represent an opportunity to promote psychoeducation on MDD and to transpose environmental enrichment programs as an adjuvant in the treatment of mood disorders in humans.

## Figures and Tables

**Table 1:** Sample characteristics. Sociodemographic and clinical variables of interest.

Parameter	Total	TDM	Control	Statistics
Age (years)	26.75	26.17 ± 2.87	27.33 ± 2.87	t = 0.4176, df = 10, *p* = 0.6851
Gender—Women/Men	4/8	2/4	2/4	—
HDRS	—	26.5 ± 6.56	4.16 ± 1.83	t = 8.025, df = 10, *p* < 0.0001
BDNF (ng/mL)	—	17.09 ± 4.59	19.58 ± 5.06	t = 0.8904, df = 10, *p* = 0.3942

Abbreviations: Hamilton Depression Rating Scale (HDRS), Brain-Derived Neurotrophic Factor (BDNF).

**Table 2:** Variables with significant associations. Those relationships that are explained within the context of the neurobiology of MDD and its relationship with the EEI are shown in bold.

Correlations		*p*	R
**HDRS**	**MDD**	**0.002**	**0.875**
**BDNF**	**CE**	**0.035**	**0.620**
**BDNF**	**COR**	**0.04**	**0.608**
**BDNF**	**EST**	**0.022**	**0.810**
**EEI**	**PE**	**<0.001**	**1.000**
AGE	LDL	0.023	0.657
AGE	COL	0.002	0.820
TSH	T3	0.038	0.612
TSH	T4	0.035	0.622
T4	GLU	0.024	0.655
TRI	GLU	0.006	0.761
LH	LDL	0.015	0.833
**SE**	**HDRS**	**0.001**	**−0.864**
**SE**	**MDD**	**0.004**	**−0.846**
HDL	TRI	0.045	−0.594

Abbreviations: Hamilton Depression Rating Scale (HDRS), Cognitive Environment (CE), Social Environment (SE), Physical Environment (PE), Major Depressive Disorder (MDD), Brain-Derived Neurotrophic Factor (BDNF), triiodothyronine (T3), thyroxine (T4), Thyroid Stimulating Hormone (TSH), High Density Lipoproteins (HDL), Low Density Lipoproteins (LDL), Total Cholesterol (COL), Triglycerides (TRI), Glucose (GLU), Morning Cortisol (COR), Luteinizing Hormone (LH).

**Table 3:** Confirmation of classification of participants by EE level.

Environmental Enrichment Domains	CS LEE	MDD LEE	CS MEE	MDD MEE	Statistics
Cognitive (points)	48.67 ± 4.04	20.33 ± 14.98	47.0 ± 1.73	51.0 ± 5.29	*p* = 0.005, F = 9.12
Social (points)	55.33 ± 7.02	30.0 ± 8.88	61.67 ± 10.41	40.67 ± 6.65	*p* = 0.006, F = 8.73
Physical Exercise (METs)	717.3 ± 157.7	5115 ± 4816	1734 ± 1051	3423 ± 1188	*p* = 0.235, F = 1.74
Environmental Enrichment Indicator (points)	821.3 ± 150.5	5165 ± 4839	1842 ± 1040	3515 ± 1190	*p* = 0.246, F = 1.68

The averages of the scores obtained and the analysis by one-way ANOVA are shown. Abbreviations: Control Subject with Low Level of Enriched Environment (CS LEE), Participant MDD with Low Level of Enriched Environment (MDD LEE), Control Subject with Medium Level of Enriched Environment (CS MEE), MDD Participant with Medium Level of Enriched Environment (MDD MEE).

## Data Availability

Data are unavailable because the participants did not agree to publish their complete information; they only accepted the publication of the study results without data that could identify them.
